# Enhancement of Electrochemical Performance by the Oxygen Vacancies in Hematite as Anode Material for Lithium-Ion Batteries

**DOI:** 10.1186/s11671-016-1783-0

**Published:** 2017-01-05

**Authors:** Peiyuan Zeng, Yueying Zhao, Yingwu Lin, Xiaoxiao Wang, Jianwen Li, Wanwan Wang, Zhen Fang

**Affiliations:** 1 Key Laboratory of Functional Molecular Solids, Ministry of Education, Center for Nano Science and Technology, College of Chemistry and Materials Science, Anhui Normal University, Wuhu, 241000 People’s Republic of China; 2 School of Chemistry and Chemical Engineering, University of South China, Hengyang, 421001 China; 3 Present address: East Beijing Road 1#, Wuhu, Anhui Province People’s Republic of China

**Keywords:** Hematite, Oxygen vacancies, Calcination, Lithium-ion batteries

## Abstract

The application of hematite in lithium-ion batteries (LIBs) has been severely limited because of its poor cycling stability and rate performance. To solve this problem, hematite nanoparticles with oxygen vacancies have been rationally designed by a facile sol–gel method and a sequential carbon-thermic reduction process. Thanks to the existence of oxygen vacancies, the electrochemical performance of the as-obtained hematite nanoparticles is greatly enhancing. When used as the anode material in LIBs, it can deliver a reversible capacity of 1252 mAh g^−1^ at 2 C after 400 cycles. Meanwhile, the as-obtained hematite nanoparticles also exhibit excellent rate performance as compared to its counterparts. This method not only provides a new approach for the development of hematite with enhanced electrochemical performance but also sheds new light on the synthesis of other kinds of metal oxides with oxygen vacancies.

**Electronic supplementary material:**

The online version of this article (doi:10.1186/s11671-016-1783-0) contains supplementary material, which is available to authorized users.

## Background

Because of its high theoretical capacity, natural abundance, and environmental friendliness, hematite (α-Fe_2_O_3_) has been regarded as a promising anode material for lithium-ion batteries (LIBs) [1–4]. However, the practical application of hematite is greatly limited because of its low conductivity, large volume variation, and easy aggregation during the discharge/charge process [5–9]. To overcome these drawbacks, two main methods are employed. The first method concerns on the synthesis of nano-sized iron oxides with different structures, which will shorten the transportation distances of electron and Li^+^. The second method focuses on elevating the conductivity of hematite, which is mainly realized by forming the composite between hematite and materials with high electronic conductivity [10–14]. Despite these progresses, a simpler method for the preparation of hematite with enhanced electrochemical performance is still needed when considering its practical uses.

The introduction of oxygen vacancies into metal oxides has been proved to be an effective method to modulate the intrinsic electrochemical properties of the metal oxides [15, 16]. The existence of oxygen vacancies could effectively change the electronic structure of the metal oxides, reduce the energy requirement for electron or ion diffusion, and lower the resistance, which could be beneficial to improve the electrochemical performances of the metal oxides [17]. What is more, previous reports also clearly indicate that the existence of oxygen vacancies could facilitate the phase transition and reduce the stress during Li^+^ insertion/depletion, which will be helpful to improve the rate performance as well as the cycling stabilities of the electrode materials. Oxygen vacancies could also provide more physical space for Li^+^ storage thus improving the specific capacity of the materials [18, 19]. For this reason, a large number of efforts have been devoted to the synthesis of electrode material with oxygen vacancies, all of which have shown enhanced electrochemical performance when used in LIBs. For example, Li_3_VO_4−δ_ was synthesized by annealing Li_3_VO_4_ powders in vacuum, and the introduction of oxygen vacancies lead to the enhanced initial coulombic efficiency, reversible capacity, and cycling stability [20]. The as-synthesized Li_3_VO_4−δ_ delivers a reversible capacity of 247 mAh g^−1^ after 400 cycles at 500 mA g^−1^, which is much higher than the corresponding value of pristine Li_3_VO_4_ (64 mAh g^−1^). MoO_3−*x*_ nanosheets were synthesized by oxidizing Mo powers in the atmosphere containing H_2_O_2_ and absolute ethanol. The as-prepared materials exhibit fascinating reversible capacity and long-term cycling stability (179.3 mAh g^−1^ at 1 A g^−1^) when used as anode materials for sodium ion batteries [18]. Anatase TiO_2−δ_–carbon nanotubes (CNTs) composites were prepared by a two-step CVD method. The CNT grown on TiO_2_ leads to the formation of oxygen vacancies under the reducing atmosphere, which greatly enhanced the electrochemical performance, especially the rate performance. The half cells cycled at 30 C can still deliver a capacity of more than 40 mAh g^−1^ [21]. V_2_O_5_ nanosheets with oxygen vacancies were also prepared by a hydrothermal reaction [22]. The as-prepared H-V_2_O_5_ electrode exhibits excellent cycling stability and improved rate capability, which could be mainly attributed to the introduction of oxygen vacancies. Tong and his co-workers proposed a facile method to generate oxygen vacancies into the materials by slight nitridation in NH_3_ atmosphere [23, 24]. Using this method, hematite and titanium dioxide with oxygen vacancies had been successfully synthesized and delivered enhanced cyclability and rate performance. Additionally, TiO_2_ heterostructured nanosheet was synthesized by hydrogenation process. This kind of heterostructured nanosheet delivered a fascinating electrochemical performance. When it was used as anode material in full battery, the full battery could achieve high energy and power density [25].

Thus, it is reasonable to believe that the electrochemical performance of hematite in LIBs could be effectively enhanced by the introduction of oxygen vacancies. However, few report concerning on the effect of oxygen vacancies in hematite has been published in the field of LIBs to date. Meanwhile, the reported method for the preparation of oxygen defect α-Fe_2_O_3_ are usually based on the thermal decomposition of FeOOH in the inert gas or in vacuum, which usually needs tedious procedure and complex equipment. In this work, we present a facile method for the synthesis of α-Fe_2_O_3_ with oxygen vacancies via a two-step process incorporating a sol–gel synthesis of the precursor and thermal annealing of the precursor in air. In this synthetic route, the precursor was synthesized by a sol–gel method and then calcined in air to yield α-Fe_2_O_3_ nanoparticles with oxygen vacancies. The partial reduction of Fe(III) during the carbon-thermic process leads to the formation of oxygen vacancies in the final product, which has also been reported for the synthesis of titanium dioxide with oxygen vacancies [21, 26]. Compared with the previous reports, the preparation of α-Fe_2_O_3_ nanoparticles with oxygen vacancies is more simple, which will lower the cost during the production process. What is more, this method can be easily scaled up by simply increasing the initial amount of the starting material. These two fascinating characteristics make this method suitable for the large-scale application in the future. Thanks to the oxygen vacancies, the electrochemical performance of α-Fe_2_O_3_ is greatly promoted. Remarkably, the as-prepared Fe_2_O_3−δ_ still maintained a reversible capacity of 1252 mAh g^−1^ at 2 C after 400 cycles. Meanwhile, the as-obtained Fe_2_O_3−δ_ also exhibit excellent rate performance. Even being cycled at 40 C, the as-prepared electrode material can still deliver a discharge capacity of 188 mAh g^−1^, which is much higher than the corresponding value than the reported α-Fe_2_O_3_ electrode material. This synthetic method not only provides a new method for the enhancement of hematite-based electrode materials but also sheds a new light for the preparation of metal oxides with oxygen vacancies.

## Methods

### Synthesis of Fe_2_O_3−δ_ Nanoparticles

In a typical procedure, 2 mmol FeCl_3_·6H_2_O and 4 mmol urea were dissolved in 46 mL distilled water with continuous stirring. Then, 4 mL acrylic acid was added into the as-formed yellow solution. In the next step, the mixed solution was transferred into a 70-mL Teflon-lined stainless steel autoclave and maintained at 140 °C for 12 h. After cooling down to room temperature, the gel-like product was collected by centrifugation, washed with distilled water and absolute alcohol several times and then dried in an oven at 80 °C overnight. To obtain hematite with oxygen vacancies, the as-formed precursor was calcined at 350 °C for 1.5 h in air with a heating rate of 2 °C min^−1^.

### Sample Characterizations

The morphology and structure of the sample was investigated by transmission electron microscopy (TEM, Hitachi HT 7700) and high-resolution TEM (HRTEM, JEOL-2010). X-ray diffraction patterns were obtained using a Bruker D8 Advance with Cu-K_α_ radiation. X-ray photoelectron spectra (XPS) of the samples were recorded on an ESCALAB 250. The thermogravimetric analysis (TGA) was carried out on SDT 2960 with a heating rate of 10 °C min^−1^ from 20 to 600 °C. The BET surface area was determined on an ASAP 2460 sorption apparatus. All the as-prepared samples were degassed at 150 °C for 10 h prior to nitrogen adsorption measurements. Electron paramagnetic resonance (EPR) tests were carried out on a Bruker A300 spectrometer (X-band, frequency 9.43 GHz) equipped with Bruker ER4141VTM liquid nitrogen system. The microwave power was 0.595 mW and modulation amplitude 3.0 G. The samples were measured at 90 K with center field 3500 G and sweep width 5000 G.

### Electrochemical Measurements

The electrochemical measurements were performed on coin-type cells (CR2032). The electrode was prepared using active material, acetylene black (Super P), and polyvinylidene fluoride (PVDF) in a weight ratio of 6:2:2. The electrolyte was a solution of 1 M LiPF_6_ in a mixture of EC:DEC (1:1 by volume). The cells were assembled in in an argon-filled glovebox (Mikrouna, Super (1220/750/900)) with both moisture and oxygen concentrations below 0.1 ppm. The galvanostatic discharge/charge characteristics were tested in the potential window of 0.01 to 3.0 V using a Neware battery tester. Cyclic voltammetry (CV) and electrochemical impedance spectroscopy (EIS) were tested on a CHI660E electrochemical workstation.

## Results and Discussion

The hydrothermal process at 140 °C for 12 h will lead to the formation of the gel-like precursor, which will be used as the starting material for the preparation of hematite with oxygen vacancies. The corresponding XRD pattern of the precursor (Additional file 1: Figure S1a) clearly indicates that the precursor is mainly composed of FeOOH (JCPDS No. 29–0713), which is obtained by the hydrolysis of Fe^3+^ in the solution. The TEM observation of the precursor (Additional file 1: Figure S1b) further confirms that the as-formed nanoparticles are wrapped in a gel-like matrix. To get further insight into the composition of the precursor, FT-IR was employed and the corresponding result is shown in Additional file 1: Figure S2. The corresponding result clearly indicates the formation of polyacrylic acid (PAA). The absorption bands centering at 1634 and 984 cm^−1^ could be attributed to the C=C and =CH_2_, respectively. And the absorption band at 1705 cm^−1^ can be assigned to the C=O double bond vibration of COOH groups [27, 28]. And the formation of PAA could be proved according to the disappearance of the absorption bands centering at 1634 and 984 cm^−1^, which has been widely reported in the previous reports [29]. Thus, the as-obtained precursor could be regarded as a nanocomposite forming by dispersing the FeOOH nanoparticles in the matrix of PAA.

**Fig. 1:**
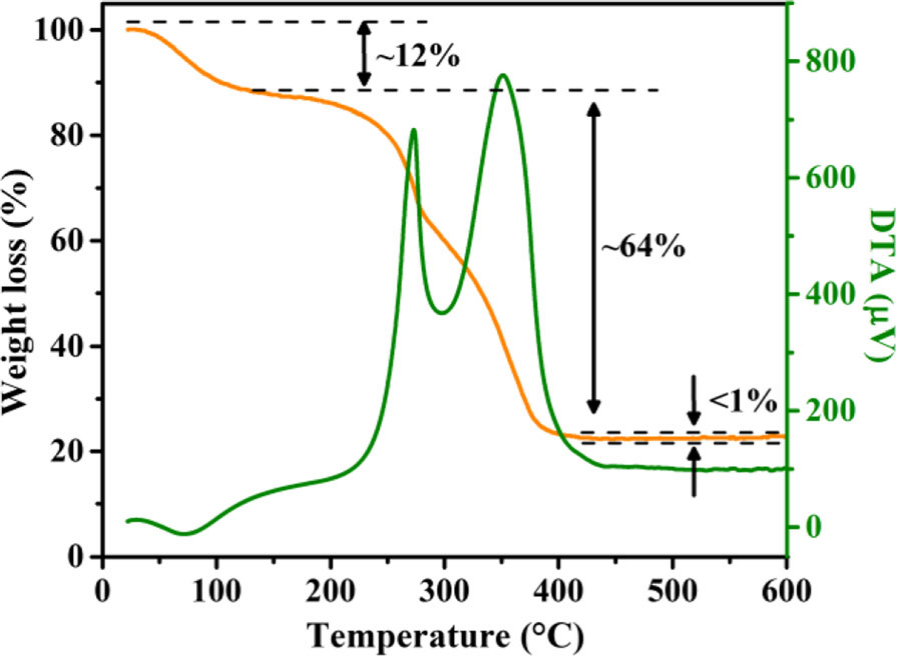
TG–DTA analysis curves of the as-prepared precursor

For the formation of hematite with oxygen vacancies, an in situ carbon-thermic process was employed. This process can be divided into two steps: (i) the carbonization process of PAA; (ii) the transformation of FeOOH to hematite and thermal reduction of hematite with the as-formed carbon in the first step. To understand good control of the in situ carbon-thermic process, thermogravimetric analysis was employed as a guide here (Fig. 1). The total weight loss during the heating process is about 76%, indicating the high content of organic compounds in the precursor. The first stage of weight loss below 150 °C is about 12%, which can be ascribed to the evaporation of water molecules in the gel-like precursor. A major weight loss can be observed in the temperature range between 200 and 400 °C, which can be ascribed to the carbonization of PAA, the decomposition of FeOOH, the partial reduction of the as-formed hematite, and combustion of the as-formed carbon, respectively. Less than 1% weight loss can be observed as the temperature is higher than 450 °C, indicating the burnout of carbon. The DTA analysis curve has two exothermic peaks locating at 273 and 350 °C. The first exothermic peak can be ascribed to the carbonization of the organic component, while the second exothermic peak may correspond to the formation of hematite and carbon-thermic reduction of hematite. During the heating progress, the corundum crucible was filled with CO_2_, which would provide a hypoxic environment and lead to the formation of α-Fe_2_O_3_ with oxygen vacancies.

**Fig. 2:**
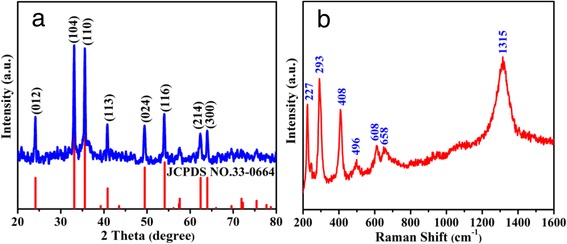
**a** XRD pattern of the obtained sample. **b** Raman spectra of Fe_2_O_3−δ_

According to the above analysis, the carbon-thermic reduction process during the calcination process may also lead to the formation of impurities such as Fe_3_O_4_ or carbon in the final product. To exclude the existence of Fe_3_O_4_ or carbon, both XRD and Raman spectra were employed. Figure 2a is the XRD pattern of the as-prepared sample, on which all the diffraction peaks can be indexed to be the rhombohedral-phased α-Fe_2_O_3_ (JCPDS No. 33–0664). No other diffraction peaks belonging to C or Fe_3_O_4_ is detected, indicating high purity of the sample. To further exclude the existence of C or Fe_3_O_4_, the Raman spectrum was employed and the result is shown in Fig. 2b. The peaks locating at 227, 293, 408, 496, 608, 658, and 1315 cm^−1^ are the typical peaks for α-Fe_2_O_3_, indicating the formation of rhombohedral-phased α-Fe_2_O_3_. The peaks centering at 227 and 496 cm^−1^ correspond to the A_1*g*_ modes of α-Fe_2_O_3_, while the peaks centered at 293, 408, and 608 cm^−1^ can be assigned to the E_g_ modes of α-Fe_2_O_3_ [30]. The peak locating at 658 cm^−1^ could be attributed to the disorder effects or the presence of hematite nanocrystals [31, 32]. The broad peak centered at 1315 cm^−1^ can be assigned to the two-magnon scattering which results from the interaction between two magnons [33]. No peaks can be found at 1350 and 1580 cm^−1^, which indicates the absence of carbon in the sample [34]. Meanwhile, the typical Raman peaks for Fe_3_O_4_ are also not detected, indicating that the as-obtained sample is α-Fe_2_O_3_.

**Fig. 3:**
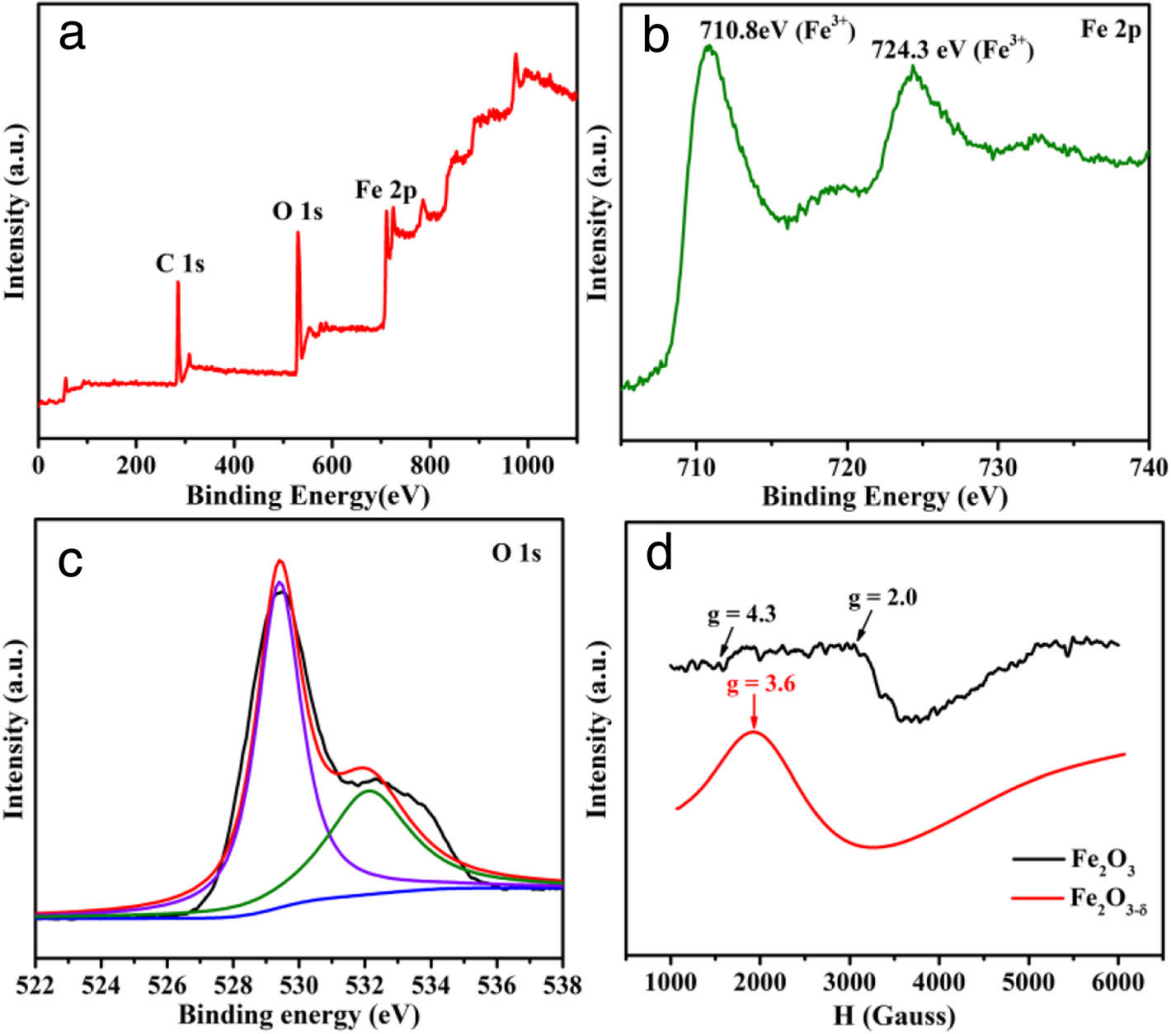
**a** The wide-survey, **b** Fe 2p, and **c** O 1s XPS spectra of Fe_2_O_3−δ_. **d** EPR spectra of Fe_2_O_3−δ_ collected at 90 K, where *H* is the magnetic field

The overall XPS spectrum of the as-prepared sample is shown as Fig. 3a, which clearly reveals the presence of oxygen vacancies in the as-obtained product. As it is shown in Fig. 3b, the peak centering at 710.8 and 724.3 eV are the characteristic peaks of Fe^3+^ in hematite [35–38]. The existence of oxygen vacancies in the as-prepared sample can be proved according to the XPS spectrum of O1s (Fig. 3c). The peak locating at 529.4 eV could be ascribed to the lattice oxygen of hematite, while the peak centering at 532.1 eV is associated with the oxygen vacancies in hematite [39–42]. To further confirm the existence of oxygen vacancies in the as-prepared materials, EPR spectra of Fe_2_O_3−δ_ was employed (Fig. 3d). For comparison purpose, the EPR spectrum of the commercial α-Fe_2_O_3_ is also recorded. As it is shown in Fig. 3d, the commercial α-Fe_2_O_3_ shows EPR signals at *g* = 2.0 and *g* = 4.3, which could be attributed to Fe^3+^ ions coupled by exchange interactions and Fe^3+^ ions in rhombic and axial symmetry sites, respectively [43, 44]. Because Fe^2+^ ions are not directly involved in the EPR absorption, it only shows a single broad resonance line centered at about *g* = 3.6. This phenomenon can be attributed to the interactions between Fe^2+^ ions and Fe^+3^ ions, which will influence the lines shape as a result. Based on the above characterizations, the existence of oxygen vacancies can be clearly proved. Thus, the chemical formula of the as-obtained product could be described as α-Fe_2_O_3−δ_ [43, 44].

**Fig. 4:**
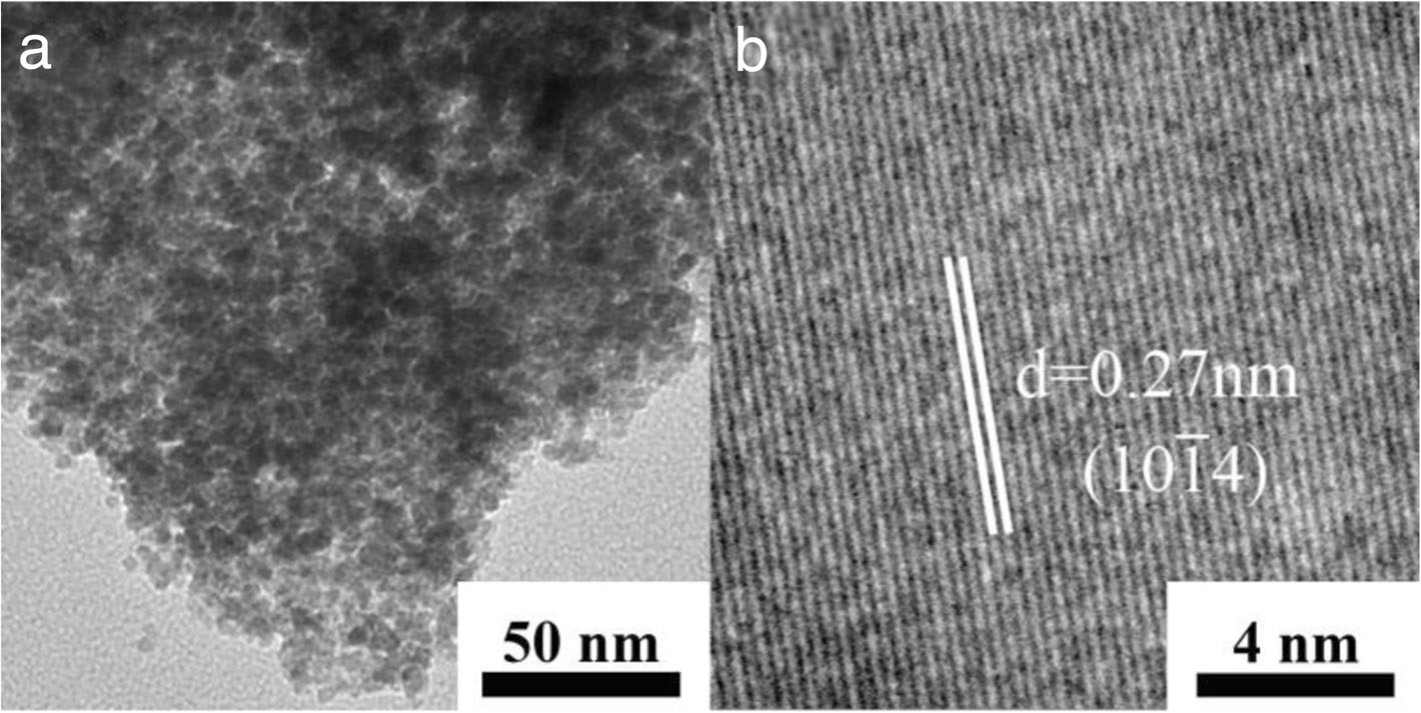
**a** TEM image of the as-prepared Fe_2_O_3−δ_ and **b** the corresponding HRTEM image

According to the TEM observation (Fig. 4a), the as-prepared sample is composed of a large number of nanoparticles, with diameters ranging from ~5 to 20 nm. Meanwhile, the as-prepared α-Fe_2_O_3−δ_ sample is mesoporous according to the TEM observation. The typical lattice distance is determined to be 0.27 nm for the as-prepared sample, which corresponds to the $\left(10\overline{1}4\right)$ lattice plane (Fig. 4b). This result further confirms that the as-prepared sample is rhombohedral phased α-Fe_2_O_3_. The porous nature of the as-prepared α-Fe_2_O_3−δ_ sample is further proved by the nitrogen adsorption–desorption experiment. The BET surface area of the as-prepared α-Fe_2_O_3−δ_ sample is determined to be 54.58 m^2^ g^−1^. And the BJH pore size distribution centers at about 6 nm, corresponding to the interspaces between these nanoparticles (Additional file 1: Figure S3).

**Fig. 5:**
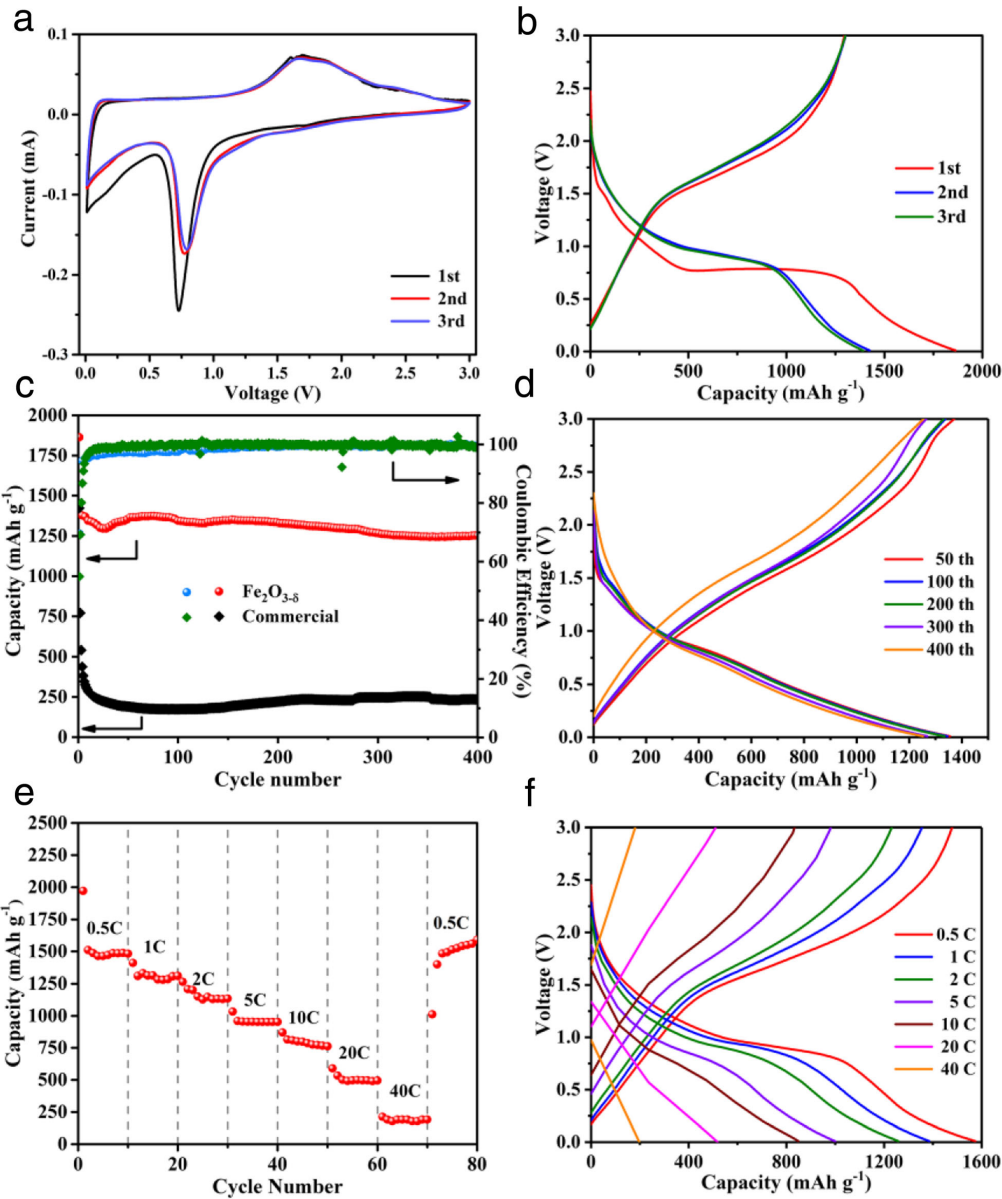
**a** CV curves of the as-prepared Fe_2_O_3−δ_. **b** The initial three galvanostatic charge/discharge profiles of Fe_2_O_3−δ_. **c** Cycling performance and coulombic efficiencies of Fe_2_O_3−δ_ and commercial Fe_2_O_3_ at 2 C. **d** Typical charge/discharge curves of Fe_2_O_3−δ_ sample during long-term cycles at 2 C. **e** Rate performance of Fe_2_O_3−δ_ at various current densities. **f** Charge/discharge curves of Fe_2_O_3−δ_ sample at different current densities

The electrochemical behavior of the as-prepared α-Fe_2_O_3−δ_ was firstly studied by the CV measurements at a scanning rate of 0.1 mV s^−1^, with the potential window from 0.01 to 3.0 V (Fig. 5a). In the first cycle, two cathodic peaks at 1.6 and 0.72 V can be observed, which corresponds to the insertion of Li^+^ into α-Fe_2_O_3−δ_ and the reduction of Fe_2_O_3−δ_ into metallic Fe. In the anodic progress, a broaden peak (between 1.6 and 1.9 V) and an ambiguous peak (at 2.3 V) can be observed, corresponding to the electrochemical oxidation reaction of metallic Fe to Fe^2+^ and Fe^3+^, respectively [34]. In the second and third cycles, the cathodic peak shifts from 0.72 to 0.79 V, and the intensity greatly decreases, which may result from the decomposition of the electrolyte and formation of the solid electrolyte interphase (SEI) layer in the first cycle [45–49]. The CV plots overlap in the following cycles, indicating the excellent reversibility of the materials. The electrochemical reaction of this process can be expressed as follows:

1 $$ {\mathrm{Fe}}_2{\mathrm{O}}_{3-\updelta} + x\ {\mathrm{Li}}^{+} + x\ {\mathrm{e}}^{-}\to {\mathrm{Li}}_x{\mathrm{Fe}}_2{\mathrm{O}}_{3-\updelta} $$

2 $$ {\mathrm{Li}}_x{\mathrm{Fe}}_2{\mathrm{O}}_{3-\updelta} + \left(2-x\right)\ {\mathrm{Li}}^{+} + \left(2-x\right)\ {\mathrm{e}}^{-}\to\ {\mathrm{Li}}_2{\mathrm{Fe}}_2{\mathrm{O}}_{3-\updelta} $$

3 $$ {\mathrm{Li}}_2{\mathrm{Fe}}_2{\mathrm{O}}_{3-\updelta} + \left(4-2\updelta \right)\ {\mathrm{Li}}^{+} + \left(4-2\updelta \right)\ {\mathrm{e}}^{-}\to\ 2{\mathrm{Fe}}^0 + \left(3-\updelta \right)\ {\mathrm{Li}}_2\mathrm{O} $$

Figure 5b is the initial three discharge/charge curves of α-Fe_2_O_3−δ_ at a current density of 2 C. The initial discharge/charge capacities for the as-prepared α-Fe_2_O_3−δ_ are 1863/1296 mAh g^−1^, respectively. According to previous reports, oxygen vacancies are easily re-oxidized over time and the high conductivity also gradually diminishes [16]. Nevertheless, the as-prepared Fe_2_O_3−δ_ sample in this work exhibits excellent cycling stability during the charge/discharge process (Fig. 5c). On the contrary, commercial Fe_2_O_3_ delivers a low initial coulombic efficiency and poor cyclability. Only about 250 mAh g^−1^ discharge capacity could be maintained after 20 cycles under identical condition. In initial several cycles, the electrodes of Fe_2_O_3−δ_ show a slight decrease in capacity, which can be ascribed to the slow formation rate of complete SEI layer at high current density. Typical charge/discharge curves of the Fe_2_O_3−δ_ sample during long-term cycles is shown in Fig. 5d. Only slight capacity decay could be found in the whole test process. And after 400 cycles at 2 C, the discharge capacity is about 1252 mAh g^−1^, which is higher than the theoretical value of hematite (1007 mAh g^−1^). The excessive capacity can be explained from several aspects. On one hand, the materials were obtained by calcination, which will lead to the formation of lattice defects in the typical nanostructure. These lattice defects will provide more active sites for Li^+^ insertion/extraction, which could improve the specific capacity of the materials [50]. On the other hand, the decomposition and reformation of the SEI layer will also lead to the increase in capacity [51], but the central aspect is that the introduction of oxygen vacancies in the materials, which will provide more physical space for Li^+^ storage, changes the intrinsic property of the sample and leads to the higher specific capacity than theoretical value.

The as-prepared α-Fe_2_O_3−δ_ also exhibits fascinating rate performance during the charge/discharge cycles when the current density increased from 0.5 to 40 C in a stepwise manner and then returned to 0.5 C (Fig. 5e). The average reversible capacities of α-Fe_2_O_3−δ_ were 1549, 1389, 1258, 995, 848, and 556 mAh g^−1^ at the discharge rate of 0.5, 1, 2, 5, 10, and 20 C, respectively. It is worth noting that the as-obtained α-Fe_2_O_3−δ_ can still deliver a reversible capacity of 198 mAh g^−1^ at a high current density of 40 C. As the current density increased, the discharge/charge plat becomes ambiguous, indicating the redox reaction mainly occurred on the surface of the electrode materials other than the inside of the material (Fig. 5f). An average discharge capacity as high as 1590 mAh g^−1^ can maintain when the current rate returned to 0.5 C. This result clearly demonstrates that the as-obtained α-Fe_2_O_3−δ_ is a good candidate for the potential application as high-rate anode materials for LIBs.

**Fig. 6:**
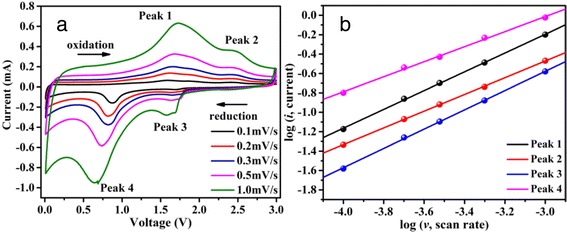
**a** CV curves at different scan rates after 50 cycles. **b** Log (*i*) versus log (*v*) plots at different redox states of the as-prepared Fe_2_O_3−δ_

To further understand the discharge/charge storage mechanism of the as-prepared materials, CV measurements of α-Fe_2_O_3−δ_ cells after 50 cycles were carried out at different scan rates, and the corresponding is shown in Fig. 6a. As the scan rates increase, the cathodic and anodic peaks shift to lower and higher potentials with increasing peak currents. The migratory peaks indicate the kinetics of Li^+^ insertion/extraction at the electrode–electrolyte interfaces. However, the increasing peak currents are not proportional to the square root of the scan rate, which indicates that the discharge/charge progresses are composed of non-Faradaic and Faradaic behavior [52–54]. And the relationship between peak current (*i*) and scan rate (*v*) can be expressed as follows:

4 $$ i = a{v}^b $$

5 $$ \log (i) = \mathrm{blog}(v) + \log (a), $$

where *i* is the peak current, *v* is the scan rate, and *a* and *b* are the adjustable parameters. The type of discharge/charge progresses can be determined by the value of *b*. When *b* = 1, the progresses mainly rely on pseudo-capacitive control, and when *b* = 0.5, the progresses are dependent on ionic diffusion. The linear relationship between log (*i*) and log (*v*) is shown in Fig. 6b. The *b* values (the slopes of the four lines) of the four redox peaks are 0.97, 0.86, 0.99, and 0.77, which means the electrochemical reactions of α-Fe_2_O_3−δ_ are controlled by pseudo-capacitive behavior. The result is in good accordance with the cycling performance. And it can also be employed to explain the reason why α-Fe_2_O_3−δ_ has a high reversible specific capacity even cycled at 2C.

The EIS of the electrodes were performed to illustrate the effect of oxygen vacancies in sample α-Fe_2_O_3−δ_. The Nyquist plots of the electrodes before cycling and after 400 cycles are shown in Additional file 1: Figure S4 with a frequency ranging from 100 to 0.01 H_z_. The Nyquist plots are composed of semicircle in the high-to-middle frequency regions and a sloping long line in the low frequency region. The smaller diameter of the semicircle indicates lower contact resistance and charge transfer resistance. The more sloping long line indicates faster kinetics during cycles. Compared with the commercial hematite, Fe_2_O_3−δ_ delivers a lower contact resistance and charge transfer resistance. This mainly ascribes to the introduction of oxygen vacancies, which could be regarded as electron donor, change the electronic structure, and facilitate the Li^+^ ion diffusion and electron transportation. After 400 cycles, the diameter of the semicircle became smaller and the long line became more sloping, which indicated the lower resistance and faster ion diffusion rate. This phenomenon may be ascribed to the irreversible reaction during discharge/charge progress, which will lead to the formation of metallic Fe or the activation of the electrode material and the formation of channels for the diffusion of lithium ions [55, 56]. Moreover, the existence of oxygen vacancies in the materials also could suppress the formation of insulated Li_2_O, which will lower the resistance.

## Conclusions

In conclusion, α-Fe_2_O_3−δ_ nanoparticles with oxygen vacancies were successfully synthesized by a two-step method incorporating a sol–gel process and following calcination of the precursor. The introduction of oxygen vacancies into hematite exerts positive impact on the electrochemical performance of the final product. The as-prepared α-Fe_2_O_3−δ_ shows enhanced electrochemical performance and cycling stability when being used as anode materials for LIBs. The existence of oxygen vacancies not only provides more space for Li^+^ storage but also facilitates the transformation of electronic structure. Meanwhile, the introduction of oxygen vacancies could also lower the contact resistance and charge transfer resistance during the discharge/charge process, leading to the enhanced electrochemical performance of the sample.

## Additional File

Additional file 1: Fig. S1. Typical XRD pattern of the precursor (a) and the corresponding TEM image (b). **Fig. S2** FT-IR spectra of the acrylic acid monomer and as-prepared precursor. **Fig. S3** Nitrogen adsorption−desorption isotherm and the corresponding pore size distribution (inset) of the as-prepared Fe_2_O_3−δ._ **Fig. S4** Nyquist plots of Fe_2_O_3−δ_ and commerical Fe_2_O_3_ before cycling and after 400 cycles at 2 C in the frequency range from 100 kHz to 0.01 Hz. (DOCX 3150 kb)
