# Supplementary material for: Enhancement of Electrochemical Performance by the Oxygen Vacancies in Hematite as Anode Material for Lithium-Ion Batteries
Source: Nanoscale Res Lett. 2017 Jan 5;12:13. doi: 10.1186/s11671-016-1783-0 (PMC5216016; doi:10.1186/s11671-016-1783-0)
Supplement: Additional file 1: Fig. S1. — Typical XRD pattern of the precursor (a) and the corresponding TEM image (b). Fig. S2 FT-IR spectra of the acrylic acid monomer and as-prepared precursor. Fig. S3 Nitrogen adsorption−desorption isotherm and the corresponding pore size distribution (inset) of the as-prepared Fe2O3−δ. Fig. S4 Nyquist plots of Fe2O3−δ and commerical Fe2O3 before cycling and after 400 cycles at 2 C in the frequency range from 100 kHz to 0.01 Hz. (DOCX 3150 kb) [file 11671_2016_1783_MOESM1_ESM.docx]

**Supporting Information**

**Supplementary Figures**


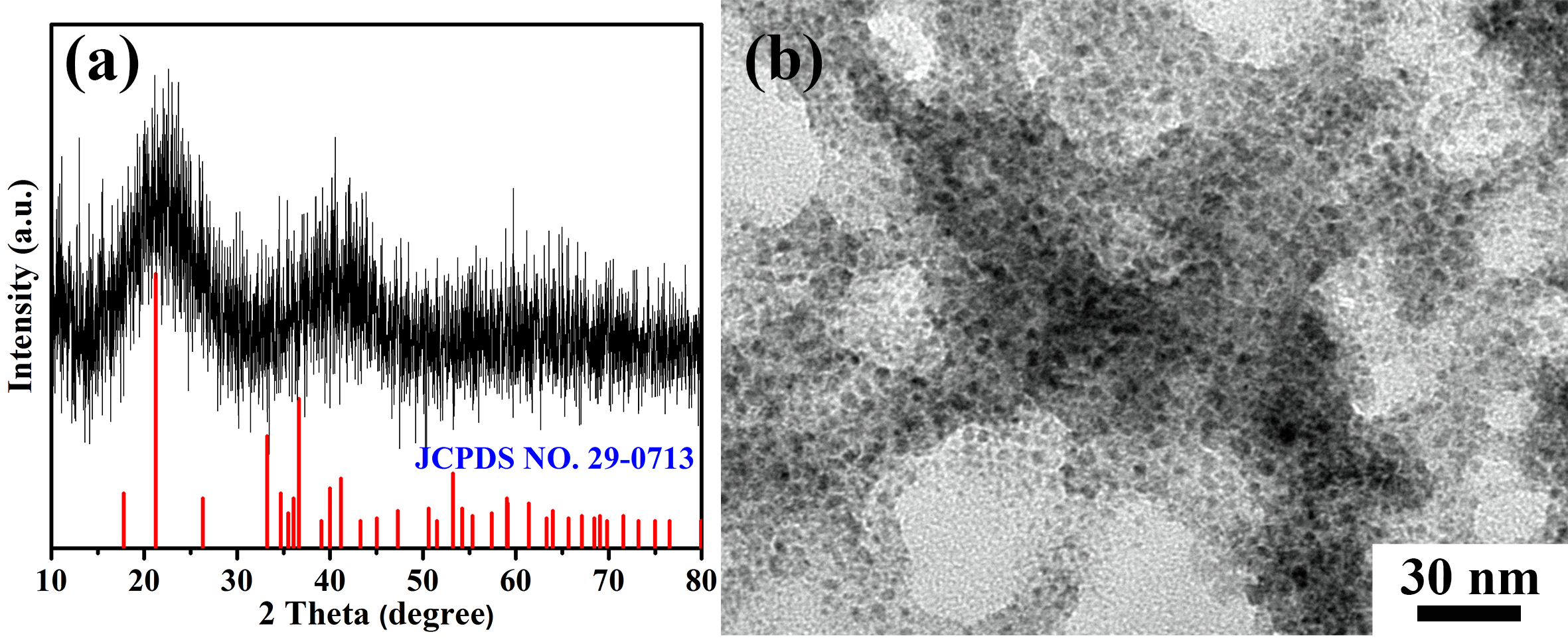


**Fig. S1** Typical XRD pattern of the precursor (a) and the corresponding TEM image (b).


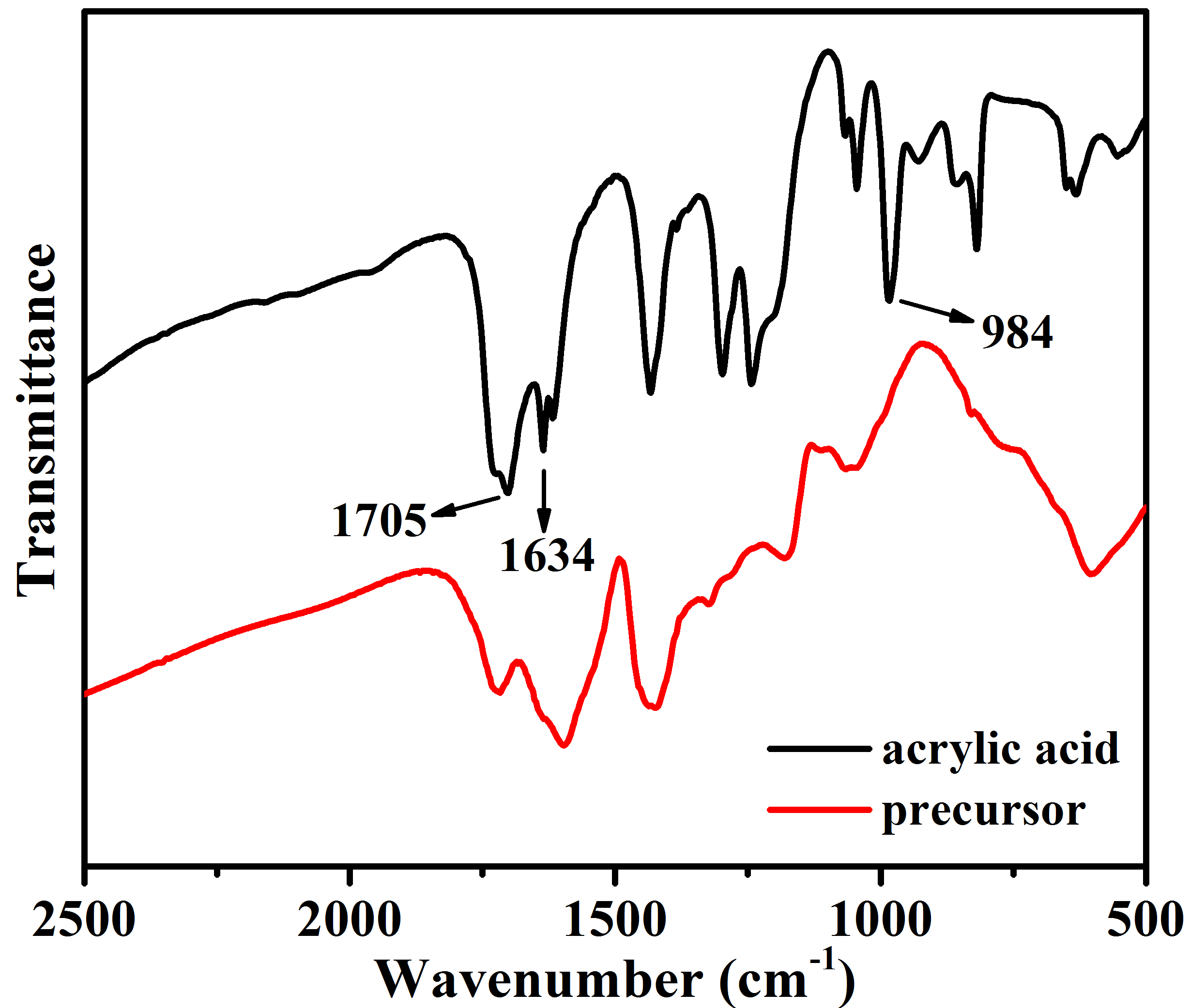


**Fig. S2** FT-IR spectra of the acrylic acid monomer and as-prepared precursor.


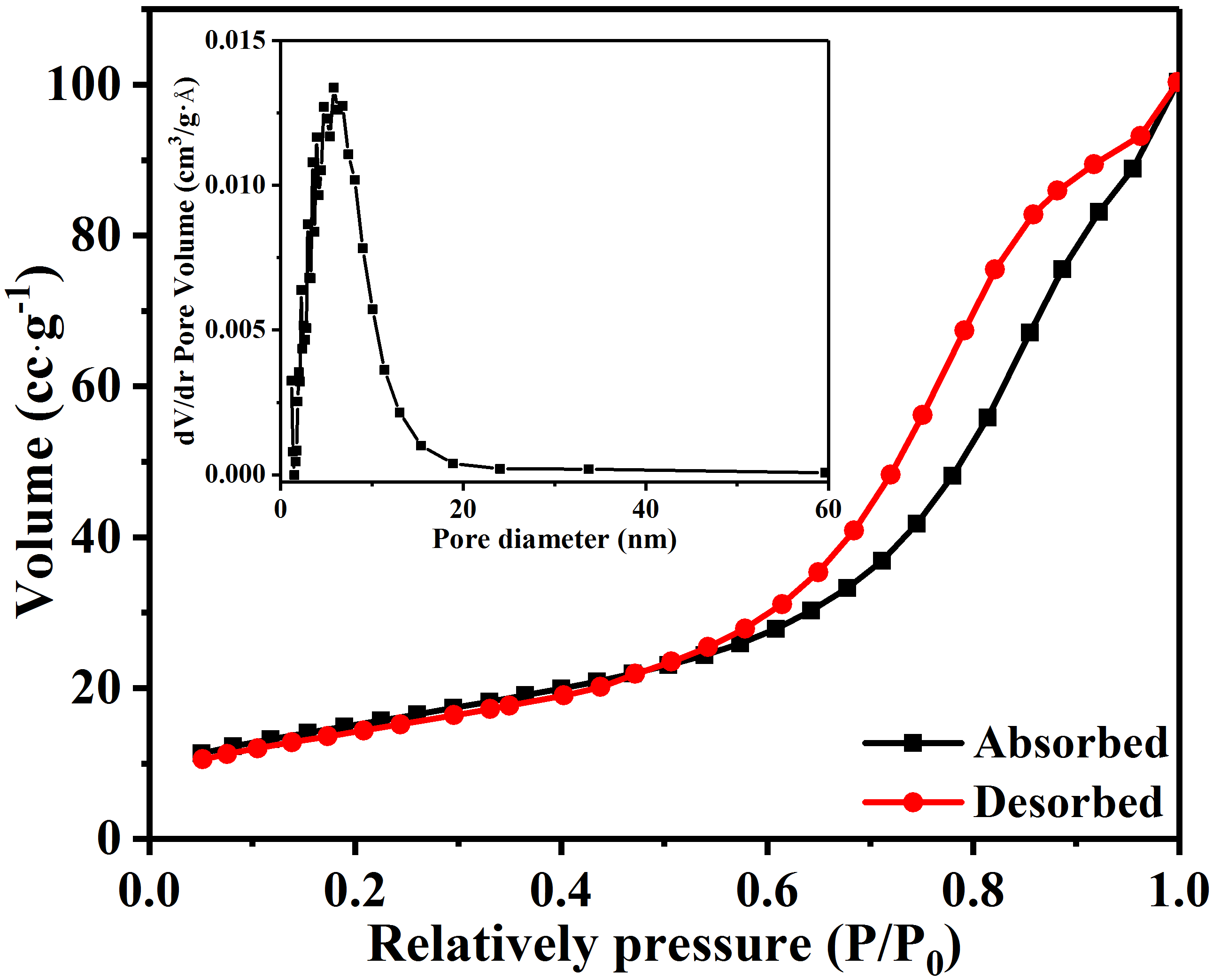


**Fig. S3** Nitrogen adsorption−desorption isotherm and the corresponding pore size distribution (inset) of the as-prepared Fe_2_O_3-δ_


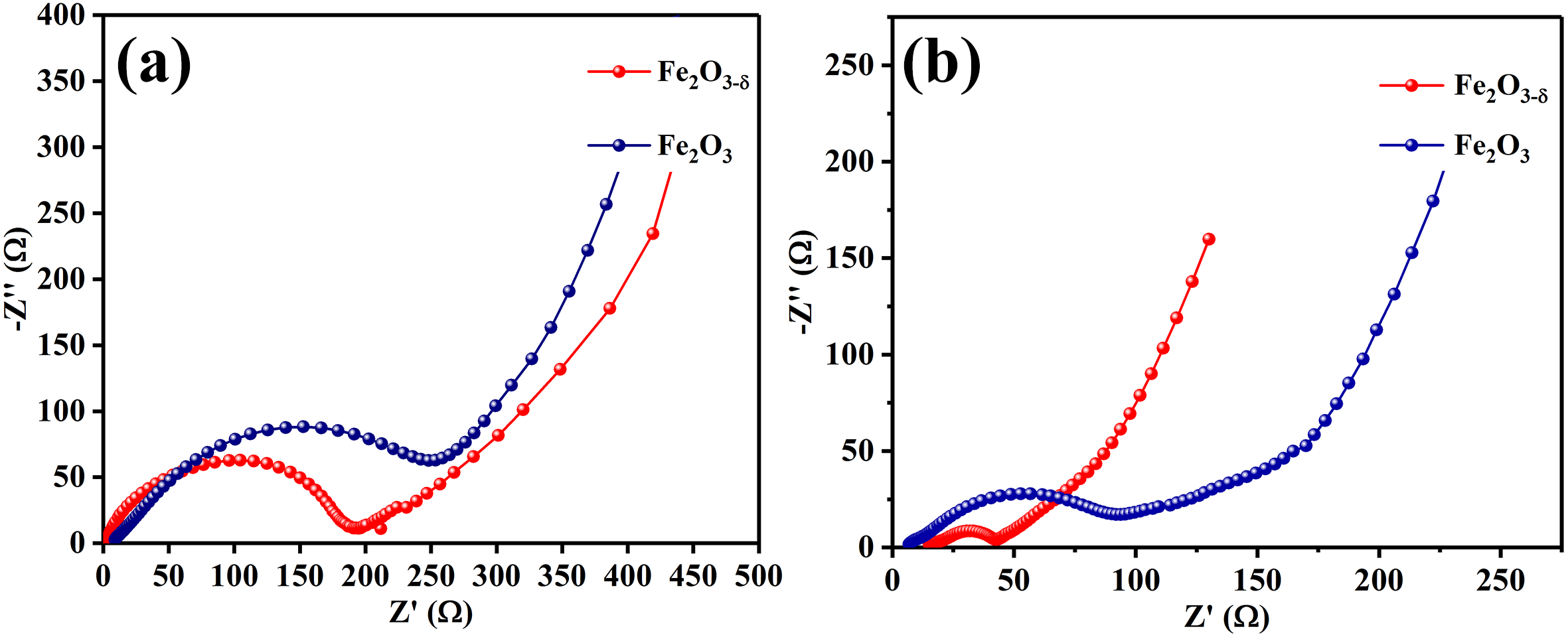


**Fig. S4** Nyquist plots of Fe_2_O_3-δ_ and commerical Fe_2_O_3_ before cycling and after 400 cycles at 2C in the frequency range from 100 kHz to 0.01 Hz.
